# Prophylactic and Therapeutic Effects of Progesterone on the Preterm Brain Injury Rat Model

**DOI:** 10.7759/cureus.70105

**Published:** 2024-09-24

**Authors:** Benson Paul, Swaminadhan D, Ananda Kumar Ponnala, Taniya Mary Martin

**Affiliations:** 1 Department of Neurology, Centre of Molecular Medicine and Diagnostics, Saveetha Institute of Medical and Technical Sciences, Saveetha University, Chennai, IND; 2 Department of Diabetes and Endocrinology, Centre of Molecular Medicine and Diagnostics, Saveetha Institute of Medical and Technical Sciences, Saveetha University, Chennai, IND; 3 Department of Anatomy, Biomedical Research Unit and Lab Animal Centre, Saveetha Dental College and Hospitals, Saveetha Institute of Medical and Technical Sciences, Saveetha University, Chennai, IND; 4 Department of Anatomy, Zebrafish Facility, Saveetha Dental College and Hospitals, Saveetha Institute of Medical and Technical Sciences, Saveetha University, Chennai, IND

**Keywords:** neurodevelopmental disorders, periventricular leukomalacia, preterm brain injury, progesterone, rat model

## Abstract

Background: Neurodevelopmental disorders are chronic conditions affecting behavior, cognition, and social function, often arising from early brain development disruptions. Preterm infants are particularly vulnerable to brain injuries, such as periventricular leukomalacia, which can lead to long-term disabilities.

Aim: This study aimed to investigate the neuroprotective effect of progesterone in reducing the severity of a preterm brain injury in rat model.

Methods: A preterm brain injury rat model was established using lipopolysaccharide to induce brain injury. The neuroprotective effects of progesterone were evaluated through histological assessments comparing treated and untreated groups. The study also aimed to assess the efficacy of progesterone in controlling brain injury severity.

Results: The findings suggest that progesterone exhibits neuroprotective properties, with a significant difference in the severity of brain injury. The histological evaluations suggest that progesterone may reduce inflammation and promote neuronal survival in preterm brain injury.

Conclusion: The administration of progesterone shows promising results as a therapeutic strategy for preventing and treating brain injuries in preterm rat pups. Further research is needed to explore the underlying mechanisms and potential clinical applications of the hormone.

## Introduction

Neurodevelopmental disorders (NDDs) are a group of chronic disorders that affect an individual in domains such as behavior, cognition, learning, and social function. NDDs are caused due to the defect in the developmental process of the brain [1]. NDDs are likely to develop at early stages of life and continue indefinitely throughout the lifetime of an affected individual. The NDDs are an expansive category of brain disruptions at a psychiatric and neurological level. NDDs comprise autism spectrum disorder, attention deficit hyperactivity disorder, cerebral palsy, intellectual disabilities, conduct disorders, and learning disabilities [2,3]. NDDs can be a result of various risk factors; some of the known factors that can lead to NDDs are genetic, immunological, trauma, hypoxic-ischemic injury, inflammation and infection, oncologic, metabolic, and iatrogenic factors [4].

Periventricular leukomalacia (PVL) is a predominant form of brain injury due to damage to the white matter near the ventricles in the brain. It is a known factor that leads to cerebral palsy [5]. PVL is a form of brain injury that is caused due to ischemia, inflammation, and infection [6]. It is estimated that approximately 5% of the infants that are born preterm, i.e., born between 25-32 weeks of gestation, are more prone to get affected by PVL. The germinal matrix hemorrhages are associated with PVL. Infants who are born preterm are prone to have germinal matrix hemorrhages that lead to hydrocephalus, which can affect the corticospinal tracts and cause spastic diplegia, which is commonly associated with PVL [7].

Advanced neuroimaging techniques, such as MRI, diffusion-weighted imaging, and proton magnetic resonance spectroscopy, have played a vital role in diagnosing PVL. Imaging modalities such as MRI have been proven helpful in diagnosing PVL early in life. Animal models have been created to study the extent of the PVL mechanism in developing NDDs. Animal models, such as those induced with CNS hypoxia to mimic the ischemic conditions that lead to PVL and models induced with bacterial lipopolysaccharide (LPS), have been used to study PVL [5]. These animal models help in understating the role of various components of the brain in the pathophysiology of PVL and also help in developing new therapeutic strategies that reduce the risk of PVL in preterm infants.

The current strategies to manage infants with PVL are to prevent preterm birth by administration of corticosteroids and by maintaining cerebral perfusion after delivery. Many drugs have been experimented for the therapeutic and prophylactic effects on PVL. Currently, no approved drug is administered for the prevention and prophylaxis of PVL. Progesterone exhibits neuroprotective properties by reducing oxidative stress, neuroinflammation, and apoptosis. This study uses a preterm brain injury rat model to focus on the therapeutic and prophylactic effect of progesterone in PVL. The prophylaxis of PVL at early stages can reduce the risk and improve the quality of life in an infant diagnosed with PVL. The drugs that reduce the later risk of PVL, i.e., cerebral palsy, can greatly improve the quality of life in patients diagnosed with PVL.

## Materials and methods

Animals

Eight Wistar rats (n = 8, with two males and six females) were procured. Animals were treated according to the Committee for Control and Supervision of Experiments on Animals guidelines. Rats were quarantined for seven days and taken for experiments. Rats were maintained in a clean 12 h:12 h dark:light cycle at 25°C room temperature. Rats were provided access to food and water ad libitum. This study was approved by the Institutional Animal Ethics Committee (IAEC), Saveetha Dental College and Hospitals (approval Number: BRULAC/SDCH/SIMATS/IAEC/09-2023/09).

Drugs

The drugs used were LPS (*Escherichia coli*, K235 purified by phenol extraction, Sigma Aldrich, Burlington, MA) and progesterone (P7556-100MG, Sigma Aldrich).

Rat breeding and weaning

The female rats were placed into the male rat's home cage in a ratio of two females to one male. The rats were provided with food and water ad libitum. The date the females were added to the male cage was noted, and the females were marked for unique identification. During the breeding period, the cages were kept clean per standard cage cleaning procedures, and the animals were monitored daily for grooming, appearance, posture, activity, and food and water intake. The female rats were checked for the vaginal plug every morning. In the morning, vaginal flushing of female rats was done with the help of a pipette containing saline (0.25 mL). Then, flushed saline was observed under a light microscope (100×) to note the stage of the estrous cycle of the rat. Also, the presence of sperm was observed. The female rats were housed individually at the end of the breeding period. The female rats placed in separate cages were provided food and water ad libitum.

PVL model

The LPS from *E. coli*, K235 (Sigma Aldrich), induced the inflammation. The group containing the pups for control (n = 8) and the treatment groups for progesterone (n = 8) were subjected to the LPS intraperitoneal route at a dose of 15 mg/kg using a 1-mL syringe equipped with a 26-gauge needle. The dose was administered on postnatal days (PNDs) 2, 4, and 6. The pups were handled minimally to avoid any rejection from the mother. During the LPS induction, the mothers were first separated from the main cage and kept in a temporary cage. The mother and the pups were monitored daily for activity, posture, food, and water intake. The pups were carefully monitored for any rejection by the mother.

Prophylactic group

The pups in the prophylactic group of progesterone (n = 8) were subjected to subcutaneous administration of progesterone (P7556-100MG, Sigma Aldrich). The dose of subcutaneous injection was 8 mg/kg using a 1-mL syringe equipped with a 26-gauge needle. The dose was given on PNDs 2, 4, and 6. Four hours after the administration of progesterone, the pups were administered with the LPS intraperitoneal route at a dose of 15 mg/kg using a 1-mL syringe equipped with a 26-gauge needle.

Treatment group

The progesterone treatment groups (n = 8) were subjected to LPS intraperitoneally at a dose of 15 mg/kg using a 1-mL syringe equipped with a 26-gauge needle. The dose was administered on PNDs 2, 4, and 6. On PNDs 8, 10, and 12, progesterone was given at a dose of 8 mg/kg subcutaneously using a 1-mL syringe equipped with a 26-gauge needle.

Neuroreflex testing

**Figure 1:**
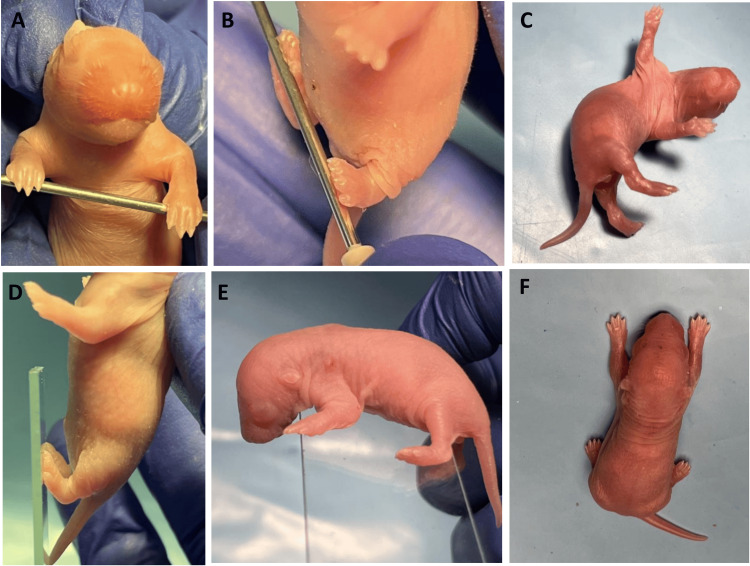
Images of reflex testing performed on PNDs 3, 4, and 6 in Wistar rat pups. (A) Forelimb grasp. (B) Hindlimb grasp. (C) Righting examination done on PND 3. (D) Hindlimb placement. (E) Cliff avoidance examination done on PND 4. (F) Gait examination done on PND 6

Neuroreflex testing started on PND 3. Each pup from all the groups was tested. Each pup was tested for all reflex tests until a positive reflex was observed (Figure 1). The positive occurrence denotes the qualification of the reflex test. No further testing was done after the positive response.

Forelimb grasping

On PND 3, the pups were tested for the forelimb grasp reflex test. A blunt cylindrical rod was placed against the forelimbs of the pups, and mild pressure was exerted. The reflex appears as a flexion of forelimb digits. The grasping is scored based on scoring levels 0-2, where score 0 is the absence of grasping, 1 is the presence of grasping on one forepaw, and 2 is the presence of grasping on both forepaws.

Hindlimb grasping

The rat pups were tested for hindlimb grasping on PND 3. A blunt cylindrical rod was placed against the hindlimbs of the pups, and mild pressure was exerted. The reflex appears as a flexion of hindlimb digits. The grasping is scored based on scoring levels 0-2, where score 0 is the absence of grasping, 1 is the presence of grasping on one hindpaw, and 2 is the presence of grasping on both hindpaws.

Righting

The righting reflex test was performed on PND 3. The rat pups were held upright. They were then let go, and the time was noted. The reflex is positive when the pups are capable of standing on all paws. The pups were given 15 seconds to complete the task. The pups were then scored based on scoring levels: 0 is lying on the back, 1 is lying on the side (left or right) or right but in the wrong posture, and 2 is achieving a successful righting.

Hindlimb placing

The pups were tested for hindlimb placement on PND 4. The torso held the pups vertically, and a flat cylindrical object gently struck the bottom of the hindpaw. The scoring of the pups was based on a scale of 0-2, where 0 indicates no reflex, 1 indicates the placing of one hindpaw (left or right), and 2 indicates the placing of both hindpaws.

Cliff avoidance

Cliff avoidance reflex testing was done on PND 4. The pups were placed on the flat edge surface so that their snouts and forepaws were over the edge. A protective reflex occurs when the pups avoid the edge. The scoring was based on a scale of 0-2, where 0 indicates the absence of a protective reflex, 1 indicates a partial attempt to avoid the edge, and 2 indicates completely moving away from the edge.

Gait

On PND 6, the pups were tested for their gait. They were placed in the center of a 15-cm circle and allowed to complete the goal in 30 seconds. Gait is achieved when the pups can move both forelimbs outside the circle in less than 30 seconds. The scoring was noted based on seconds.

Auditory startle

On PND 10, the pups were tested for auditory startle. They were presented with a loud noise directly to assess the startle response. A positive response is when the pups display a reflex or movement to the sound. The pups are scored based on no startle response (No) or positive startle response (Yes).

Posture

On PND 12, the pups were analyzed for their posture. They were placed on a flat surface to observe their posture while moving. Matured posture is achieved by lifting the abdomen from the surface or positioned parallel to the body. The pups are scored based on a scale of 0-2: 0 indicates the absence of any movements, 1 indicates partial posture while moving, and 2 indicates the presence of a matured posture.

Opening of eye

On PND 12, the opening of eyelids was analyzed [8]. The pups were scored from 0 to 2: 0 indicates eyes not visible, 1 represents any one eye is visible, and 2 indicates two visible eyes.

Morris water maze test

The pups were tested for spatial memory and learning on PNDs 31,32, & 33. The animals were pre-trained for the water maze. Pups were placed on a platform with water in the pool's center. The animals underwent three consecutive trials. First, the animals were placed on the platform for 20 seconds. Then, the animal was taken to one of the positions (north, south, east, or west) and was lowered into the water by supporting it by the hand. The animals were given 60 seconds to search for the platform. The procedure was repeated for two more trials to pretrain the animals. Each animal was then subjected to a probe trial by removing the platform. The number of crossings the animal crossed through the center was noted for a time period of 30 seconds [9].

Histology

The rats were euthanized by anesthesia overdose, and their brains were collected. The tissue samples were immersed in a 10% formalin solution. The tissues were embedded and enclosed in paraffin wax. The implanted samples were then sliced perpendicular to the brain surface to produce cross-sections of the brain with a thickness of 5mm. The sections were stained with hematoxylin and eosin (H&E). The stained sections were then examined under a light microscope to study brain tissue morphology.

Statistical analysis

The experimental data were obtained as mean ± standard error of the mean (SEM) using Jeffreys’s Amazing Statistics Program version 0.19. One-way analysis of variance (ANOVA) and Tukey’s post hoc test were used for statistical analysis, which was performed using Graph Pad version 10 software.

## Results

Neuroreflex testing

The study's main objective is to measure the efficacy of progesterone in controlling brain inflammation. The rat pups were subjected to neuroreflex testing from PND 3. The scores are noted based on a positive reflex (Table 1).

**Table 1:** Neurodevelopmental reflex outcomes Data are presented as mean ± SEM. The statistical analysis used was one-way ANOVA followed by Tukey's post hoc test ^a^Day of appearance ^b^Significant difference among the groups LPS: lipopolysaccharide; ANOVA: analysis of variance

Examination parameter	Groups (mean ± SEM)			
Neurodevelopmental reflexes	Negative control	Positive control (LPS-induced)	Prophylactic progesterone	Treatment progesterone
Forelimb grasp^a^	4.2 ± 0.2	4.9 ± 0.3	4.1 ± 0.3	4.4 ± 0.3
Hindlimb grasp^a^	4.5 ± 0.3	5.6 ± 0.3	4.9 ± 0.3	5.7 ± 0.3
Righting^a^	4.7 ± 0.2	5.5 ± 0.3	4.9 ± 0.3	5.2 ± 0.4
Hindlimb placement^a,b^	4.7 ± 0.2	6.0 ± 0.5	5.2 ± 0.3	6.7 ± 0.4
Cliff avoidance^a^	5.4 ± 0.3	6.4 ± 0.6	6.4 ± 0.5	7.4 ± 0.5
Gait^a,b^	6.1 ± 0.2	10.5 ± 0.5	9.1 ± 0.4	9.7 ± 0.2
Auditory startle^a^	10.0 ± 0.0	10.5 ± 0.3	10.5 ± 0.3	10.9 ± 0.4
Posture^a,b^	12.1 ± 0.1	13.6 ± 0.4	12.9 ± 0.2	13.2 ± 0.3
Eye-opening^a,b^	14.0 ± 0.2	15.9 ± 0.2	15.0 ± 0.2	15.9 ± 0.3

Grasping reflex

No significant difference was observed in the grasping reflex. All rat pups performed forelimb grasp on an average of 4.4 days and hindlimb grasp on an average of 5.3 days. All pups were able to perform both forelimb and hindlimb grasps.

Righting

Righting showed no significant difference among all groups. The pups were able to right on an average of 5.3 days, irrespective of the treatment given.

Hindlimb placement

The hindlimb placement showed a significant difference in the prophylactic progesterone group (5.2 ± 0.3 days, Tukey's p < 0.05) when compared to the progesterone treatment group (6.7 ± 0.4 days), where the hindlimb placement was early in progesterone prophylactic group than the treatment group suggesting the prophylactic effect of progesterone in developing brain.

Cliff avoidance

All pups could perform cliff avoidance on an average of 6.7 days. No significant differences were observed while comparing the cliff avoidance reflex among the groups.

Gait

Significant differences were observed in the gait of negative control pups (6.1 ± 0.2 days, Tukey's p < 0.0001) compared to positive control (10.5 ± 0.5 days); the positive control pups show delayed acquisition of gait. This suggests the potential of LPS in causing brain inflammation.

Auditory startle

All pups showed auditory startles for an average of 10.5 days. No significant difference was observed in this reflex.

Posture

The negative control pups (12.1 ± 0.1 days, Tukey's p < 0.05) were able to attain matured posture when compared to the positive control group (13.6 ± 0.4 days). This suggests the inflammatory potential of LPS on the developing brain.

Eye-opening

The eye-opening was attained earlier in the negative control group (14.0 ± 0.2 days, Tukey's p < 0.0001) when compared to positive control pups (15.9 ± 0.2 days). This shows the effect of LPS on exerting an inflammatory effect on the developing brain.

Morris water maze test

**Figure 2:**
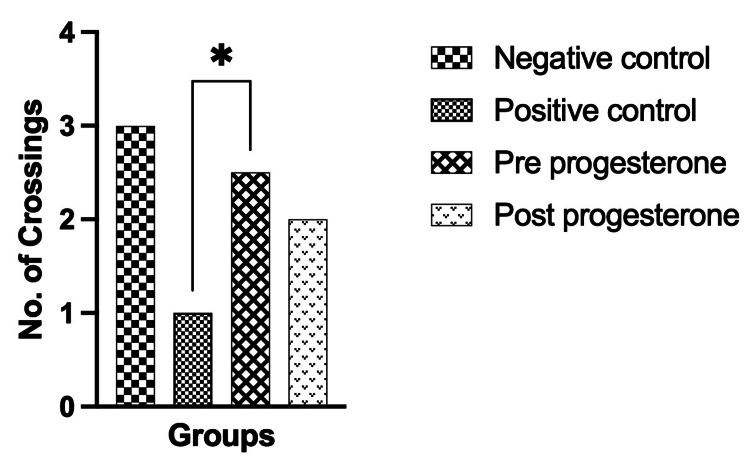
Morris water maze test, showing significant difference between positive control and prophylactic progesterone group * indicates p < 0.05

The results show better spatial memory in the prophylactic progesterone group (p < 0.05) compared to the positive control group. The progesterone prophylactic group showed better spatial memory than the treatment group (Figure 2). This shows the potential of progesterone as a prophylactic agent.

LPS-induced brain inflammation in rat pups

**Figure 3:**
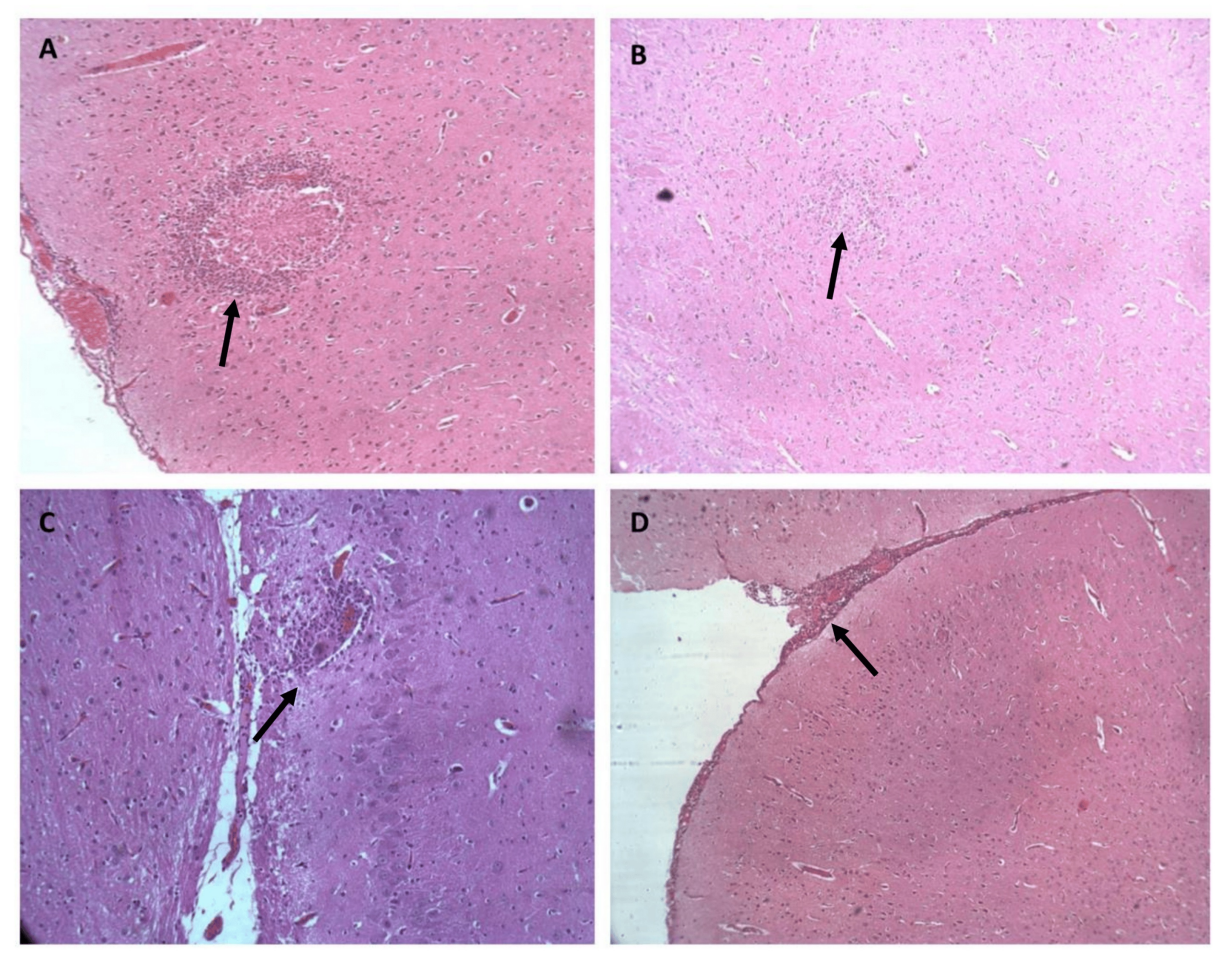
Photomicrograph of PVL positive control brain, showing (A) inflammatory cell infiltration, (B) gliosis, (C) perivascular cuffing, and (D) mononuclear cell infiltration (black arrows) PVL: periventricular leukomalacia

The rat pups induced with LPS, which were taken as a positive control, were subjected to histological analysis. The brain tissue samples were analyzed for inflammation (Figure 3). The brain samples of the rat pups revealed inflammatory cell infiltration, gliosis, perivascular cuffing, and mononuclear cell infiltration. These findings significantly suggest that LPS induced inflammatory damage to the developing brains of the rat pups.

Progesterone showed reduced brain lateral ventricle dilation

**Figure 4:**
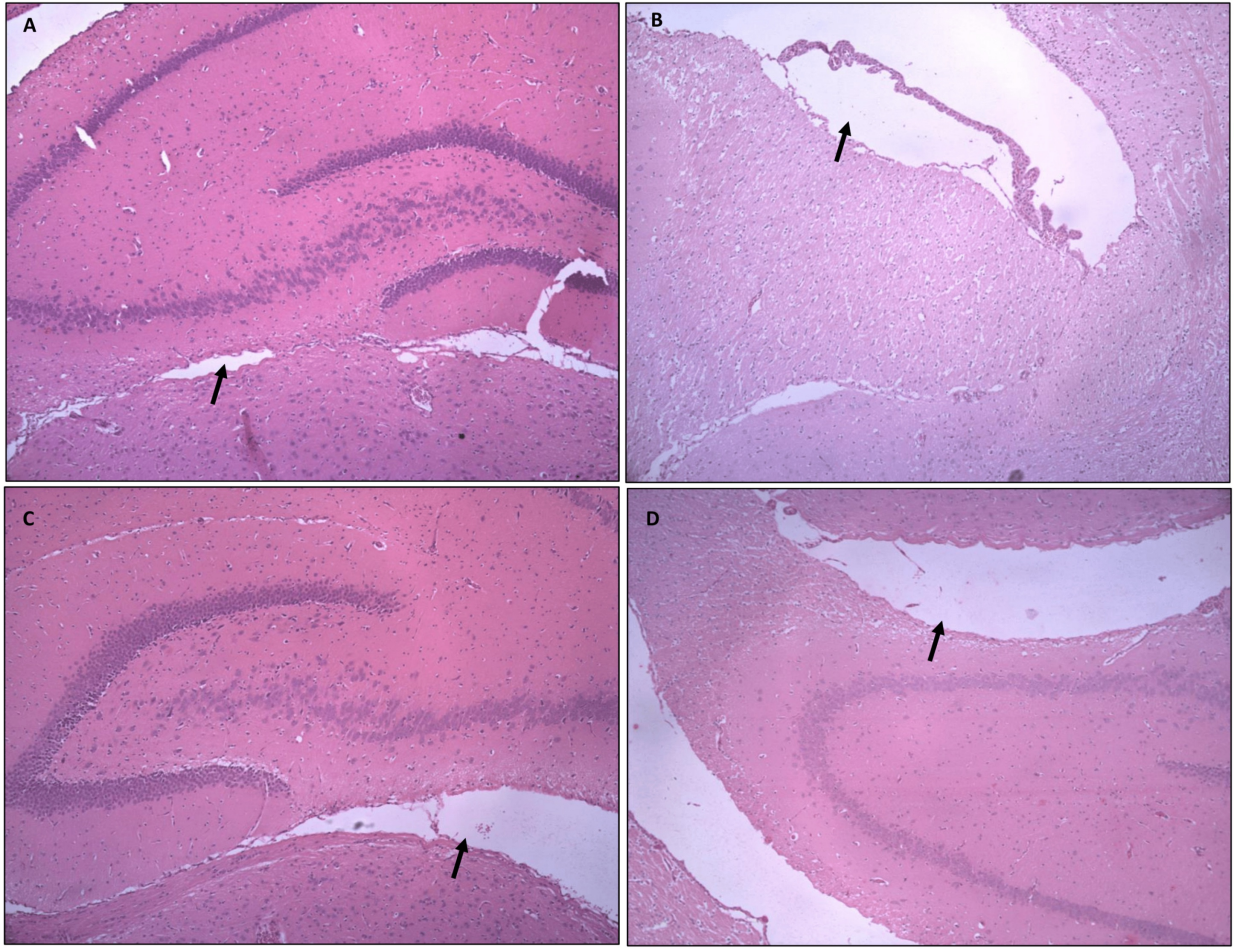
Photomicrograph of the rat pup brain showing lateral ventricle dilation in (A) negative control, (B) positive control, (C) prophylactic progesterone group, and (D) treatment progesterone group (black arrows)

Histological examination of H&E-stained sections from the rat pup brains revealed notable differences in the lateral ventricle dilation (Figure 4). The results show that the prophylactic and treatment groups of progesterone demonstrated a significant reduction in the lateral ventricle dilation compared to the positive control group. This indicates that progesterone has potential prophylactic and therapeutic effects on the brain, as well as its efficacy in reversing lateral ventricle dilation.

## Discussion

Progesterone is a steroid hormone that controls numerous physiological functions, including reproduction, neuroprotection, and neurodevelopment. Progesterone has recently gained attention for its potential therapeutic applications in NDDs and neuroinflammatory diseases. Progesterone's neuroprotective properties result from its ability to control inflammation, reduce oxidative stress, and promote myelination and neuronal development. Several studies have found that progesterone improves outcomes in traumatic brain injuries, stroke, and neurodegenerative disorders by decreasing neuronal damage and increasing functional recovery. Previous research has shown that progesterone can reduce the effects of neuroinflammatory insults in developing brains. In neonatal models, progesterone has been shown to reduce inflammatory cytokine production, protect against excitotoxicity, and promote neuronal survival and repair following injury or inflammation [10,11]. Progesterone has been shown to improve neurogenesis and reduce apoptosis in neonatal hypoxic-ischemic encephalopathy, a condition characterized by decreased blood and oxygen flow to the brain, which leads to better neurodevelopmental outcomes. Furthermore, in perinatal brain injury models, progesterone administration has been linked to reduced white matter injury and improved motor and cognitive functions, implying that it may be useful in preventing long-term neurodevelopment deficits [12]. Despite these positive results, the role of progesterone in neonatal brain inflammation caused by LPS has not been thoroughly investigated. LPS has been shown to elicit strong inflammatory responses and is commonly used as a model for studying inflammation-induced brain injury in neonates. LPS exposure during critical periods of brain development can result in severe neuroinflammation, including microglial activation, increased proinflammatory cytokine release, and neuronal damage, leading to long-term NDDs [13].

This study aims to demonstrate progesterone's prophylactic and therapeutic potential in reducing brain inflammation and associated developmental outcomes in rat pups administered with LPS to induce brain inflammation. The findings show that progesterone, when administered prophylactically, can control certain neurodevelopmental deficits that are associated with preterm brain injury. The neuroreflex results suggest varied effects of progesterone in the developing brain. The findings suggest that the grasping, righting, cliff avoidance, and auditory startle reflexes were not affected by the LPS induction. However, significant differences were observed in hindlimb placement, with the prophylactic progesterone group showing earlier acquisition of hindlimb placement (5.2 ± 0.3 days) compared to the treatment group (6.7 ± 0.4 days), suggesting that early administration of progesterone may improve the neurodevelopmental outcomes potentially by its neuroprotective and anti-inflammatory effects. The gait acquisition was significantly delayed in the positive control (10.5 ± 0.5 days) compared to the negative control group (6.1 ± 0.2 days), suggesting the impact of LPS in inducing brain inflammation. The earlier acquisition of matured posture in negative control pups (12.1 ± 0.1 days) compared to the positive control group (13.6 ± 0.4 days) and delayed eye-opening in the positive control group (15.9 ± 0.2 days) compared to the negative control group (14.0 ± 0.2 days) further supports the inflammatory role of LPS on developing brain. This result is consistent with the previous study done in an induced rat model, where they investigated the role of diet in improving the neurodevelopmental outcome of LPS-induced rat pups [8].

The Morris water maze test results show that the prophylactic progesterone group rat pups demonstrated increased spatial memory performance compared to the positive control group (p < 0.05) and treatment group. This suggests that prophylactic progesterone administration may enhance spatial memory in developing brains exposed to inflammatory conditions. This parallels the study investigating the spatial memory of preterm model rat pups, which showed increased spatial memory after treatment [14]. Histological examination of brain tissue samples from rat pups in the positive control group showed inflammatory cell infiltration, gliosis, perivascular cuffing, and mononuclear cell infiltration, consistent with LPS-induced inflammatory damage. These markers confirm the efficacy of LPS in inducing brain inflammation. Progesterone showed a significant reduction in lateral ventricle dilation compared to the positive control group when given prophylactic and as treatment. This suggests that progesterone may contribute to the anti-inflammatory and neuroprotective effects in the developing brain. This result is similar to the study that showed the effect of progesterone in preventing white matter injury in the preterm injury rat model and the neuroprotective effect of progesterone in the developing brain [15,16]. Overall, findings from this study suggest that progesterone has both prophylactic and therapeutic potential in controlling neuroinflammation and associated neurodevelopmental impairments in rat models of preterm injury.

However, this study has limitations, such as the limitations of the animal models. The use of rat models may not fully replicate the neurodevelopmental process of the human brain. Also, the brain developmental stages of rat pups may not correspond to the neurodevelopmental stages of human infants. There are limitations in the timing of progesterone administration. This study suggests that early administration of progesterone is beneficial, but the effects of varying the timing of treatment relative to LPS exposure are not explored.

## Conclusions

This study demonstrated the role of progesterone as a potential prophylactic and therapeutic agent. Progesterone has significant potential in reducing brain inflammation and neurodevelopmental outcomes. The findings showed that the prophylactic progesterone group achieved earlier acquisition of hindlimb placement, enhanced gait, and improved spatial memory compared to positive control and treatment groups. Histological analysis further showed that progesterone reduced lateral ventricle dilation, indicating its neuroprotective and anti-inflammatory effects on the developing brain. Other developmental reflexes like posture and eye-opening were delayed in the LPS-induced group supporting the inflammatory impact of LPS on brain development. However, the findings are limited to animal models and timing of administration. Further research to explore the applicability of progesterone in human preterm infants needs to be evaluated. Overall, progesterone shows promise as a prophylactic and therapeutic agent in reducing neuroinflammation and improving neurodevelopmental outcomes in conditions mimicking brain injury.
